# Applying quantum approximate optimization to the heterogeneous vehicle routing problem

**DOI:** 10.1038/s41598-024-76967-w

**Published:** 2024-10-25

**Authors:** David Fitzek, Toheed Ghandriz, Leo Laine, Mats Granath, Anton Frisk Kockum

**Affiliations:** 1 Department of Microtechnology and Nanoscience, Chalmers University of Technology, 412 96 Gothenburg, Sweden; 2 Volvo Group Trucks Technology, 405 08 Gothenburg, Sweden; 3 Department of Mechanics and Maritime Sciences, Chalmers University of Technology, 412 96 Gothenburg, Sweden; 4 Department of Physics, University of Gothenburg, 412 96 Gothenburg, Sweden

**Keywords:** Quantum information, Quantum physics

## Abstract

Quantum computing offers new heuristics for combinatorial problems. With small- and intermediate-scale quantum devices becoming available, it is possible to implement and test these heuristics on small-size problems. A candidate for such combinatorial problems is the heterogeneous vehicle routing problem (HVRP): the problem of finding the optimal set of routes, given a heterogeneous fleet of vehicles with varying loading capacities, to deliver goods to a given set of customers. In this work, we investigate the potential use of a quantum computer to find approximate solutions to the HVRP using the quantum approximate optimization algorithm (QAOA). For this purpose we formulate a mapping of the HVRP to an Ising Hamiltonian and simulate the algorithm on problem instances of up to 21 qubits. We show that the number of qubits needed for this mapping scales quadratically with the number of customers. We compare the performance of different classical optimizers in the QAOA for varying problem size of the HVRP, finding a trade-off between optimizer performance and runtime.

## Introduction

Devices utilizing quantum-mechanical effects provide a new computational paradigm that enables novel algorithms and heuristics^1–4^. The ongoing development of such devices^5–8^ provides an opportunity to test these algorithms on small problem instances, which could lead to new solutions to hard optimization problems. In this work, we show how a quantum approximate optimization algorithm (QAOA)^9^ can be employed to find approximate solutions for the HVRP^10^. Our approach can be utilized on both noisy intermediate-scale quantum (NISQ)^11^ computers and quantum annealers^12^. It also paves the way for implementing challenging instances of the HVRP on larger quantum computers in the future.

The HVRP belongs to the extensively studied class of optimization problems known as the vehicle routing problem (VRP)^13,14^ in the field of logistics. The VRP is finding the optimal set of routes for a fleet of vehicles to travel in order to deliver goods to a given set of customers. This problem is also found in supply-chain management and scheduling^15,16^. Variants of the VRP include the capacitated vehicle routing problem (CVRP), in which the vehicles have a limited carrying capacity^13^, and the HVRP studied here, in which the vehicles that are to be deployed need to be determined from a fixed set of vehicles and capacity constraints are given^10,17^. All these VRPs are challenging since they belong to the complexity class NP-hard^18^.

Due to its industrial relevance, there has been tremendous effort devoted to finding good approximate solutions to the VRP and its variants through various heuristics^19,20^, e.g., construction heuristics^21,22^, improvement heuristics^23^, and metaheuristic top-level strategies^24^. In construction heuristics, e.g., the Clarke and Wright savings algorithm^25^, one starts from an empty solution and iteratively extends it until a complete solution is obtained. In improvement heuristics, one instead starts from a complete solution (often generated by a construction heuristic) and then try to improve further through local moves. There are several software libraries and tools that implement ready-to-use solvers for all these methods^26–28^. Moreover, exact methods for solving the VRP and its variants have also been investigated^29^.

In this article, we instead investigate a heuristic method for solving the HVRP on a quantum computer. Such devices, including both programmable quantum processors^2^ and quantum annealers^12^, are gradually becoming available due to recent advances in controlling quantum systems. The current programmable gate-based quantum computers known as NISQ devices^11^ are largely limited by their intermediate number (several tens or hundreds^5,7,8,30^) of controllable qubits, limited connectivity, imperfect qubit control, short coherence times, and minimal error correction. On the other hand, quantum annealers have been able to leverage thousands of qubits, and have been successfully used to solve larger combinatorial problem instances^31^. However, only a subset of quantum algorithms can run on NISQ devices^4,32^; other algorithms require more advanced hardware.

The heuristic method we apply to the HVRP here is an example of a variational quantum algorithm (VQA)^3^, which is a promising class of hybrid quantum-classical algorithms that are compatible with NISQ devices. From a description of the problem, the first step is to define a cost (or loss) function *C*, which encodes the quality of a solution to the problem. Next, one proposes an ansatz, i.e., a quantum operation depending on a set of continuous or discrete parameters $\varvec{\theta }$. This ansatz is then optimized in a hybrid quantum-classical loop to approach $\varvec{\theta ^*} =\underset{\varvec{\theta }}{\text {argmin }} C(\varvec{\theta })$. Such algorithms have emerged as a leading contender for obtaining quantum advantage^3^ within the constraints of NISQ devices. Variational quantum algorithms have been proposed for numerous applications envisioned for quantum computers, e.g., in chemistry, logistics, and finance^33–35^.

The type of VQA we employ here is the QAOA^9^, which is a heuristic that can approximate the solution to many combinatorial problems, including VRPs. Current research in this area^36^ ranges from applications on large-scale VRP instances with a quantum annealer^37^ to more specific variants of the VRP, such as the CVRP^31^, the multi-depot capacitated VRP^38^, and the VRP with time windows^39^. These approximation algorithms have been tested on quantum annealers^31^ and NISQ devices^40^. There have also been several experimental realizations of the QAOA applied to other optimization problems^41–44^, including the travelling salesperson problem (TSP)^45^, which is of fundamental importance for VRPs.

However, a problem description suited for the QAOA, an Ising Hamiltonian^46–48^ (describing the energy of interacting two-level systems), seems to be lacking for the case of the HVRP. In this work, we provide such a mapping for the HVRP, which can be utilized on both NISQ computers and quantum annealers. We show that, in this formulation, the number of qubits scales quadratically with the number of customers. To explore the performance of QAOA applied to the HVRP, we simulate problem instances with up to 21 qubits, which corresponds to three customers and two trucks. We check how the solution quality depends both on the choice of classical optimizer and on the depth of the quantum circuit. This work lays the foundation for finding approximate solutions to large problem instances of the HVRP when sufficiently advanced quantum-computing hardware becomes available.

The paper is organized as follows. We first introduce the HVRP and its mathematical formulation. Then we develop the Ising formulation of the HVRP. Thereafter, we review the QAOA and describe how it can be used to find approximate solutions to the HVRP. We then present numerical results from applying the QAOA to a few HVRPs of different sizes. Finally, we conclude the paper and give an outlook for future work.

## The heterogeneous vehicle routing problem

The HVRP can be formulated as follows, as originally proposed in Ref.^10^ inspired by earlier works^49,50^. A fleet of vehicles is available at a depot, which becomes node 0 of a complete graph $\mathscr {G} = (\mathscr {N}, \mathscr {E})$ (we do not consider multiple depots). Here, $\mathscr {N} = \{ 0,\ldots, n \}$ is the set of nodes or vertices, such that the *n* customers that the fleet of vehicles should deliver goods to constitute the customer set $\mathscr {N}_0 = \mathscr {N} \setminus \{0\}$, and $\mathscr {E} = \{(i, j) :0 \le i, j \le n, i \ne j\}$ denotes the set of arcs. Each customer *i* has a positive demand $q_i$.

The set of available vehicle types is $\mathscr {V} = \{1,\ldots,k \}$, with $m_v$ vehicles of type $v \in \mathscr {V}$. When using these vehicles to deliver goods to meet the customer demand, several costs and constraints need to be taken into account. There is the fixed vehicle cost $t^v$, i.e., the cost that is independent of the distance travelled by a vehicle of type *v*. Then, there is the vehicle capacity $Q^v$. Different vehicle types can have the same capacities, but differ in, e.g., the type of powertrain used^17^. Finally, there is the cost $c_{ij}^v$ of travelling on edge (*i*, *j*) with a vehicle of type *v*. To describe the constraints, it is useful to introduce the binary variables $x^v_{ij}$, which are equal to 1 if and only if a vehicle of type *v* travels on edge (*i*, *j*). We denote by $f_{ij}^v$ the amount of goods that are leaving node *i* to go to node *j* using a truck of type *v*, while the amount of goods entering the node is denoted $f_{ji}^v$.

Using this notation, the HVRP is to minimize the cost

1 $$\begin{aligned} C_{\textrm{tot}} = \sum _{v \in \mathscr {V}} \sum _{j \in \mathscr {N}_0} t^v x^v_{0j} + \sum _{v \in \mathscr {V}} \sum _{(i,j) \in \mathscr {E}} c^v_{ij} x^v_{ij}, \end{aligned}$$

subject to the constraints

2 $$\begin{aligned}&\sum _{j \in \mathscr {N}_0} x^v_{0j} \le m_v \quad v\in \mathscr {V} \, , \end{aligned}$$

3 $$\begin{aligned}&\sum _{v \in \mathscr {V}} \sum _{j \in \mathscr {N}} x^v_{ij} = 1 \quad i \in \mathscr {N}_0 \, , \end{aligned}$$

4 $$\begin{aligned}&\sum _{v \in \mathscr {V}} \sum _{i \in \mathscr {N}} x^v_{ij} = 1 \quad j \in \mathscr {N}_0 \, , \end{aligned}$$

5 $$\begin{aligned}&\sum _{j \in \mathscr {N}_0} x^v_{j0} = \sum _{j \in \mathscr {N}_0} x^v_{0j} \quad v\in \mathscr {V} \, , \end{aligned}$$

6 $$\begin{aligned}&\sum _{v \in \mathscr {V}} \sum _{j \in \mathscr {N}} f^v_{ji} - \sum _{v \in \mathscr {V}} \sum _{j \in \mathscr {N}} f^v_{ij} = q_i \quad i \in \mathscr {N}_0 \, , \end{aligned}$$

7 $$\begin{aligned}&q_j x_{ij}^v \le f^v_{ij} \le (Q_v - q_i)x_{ij}^v \quad (i,j) \in \mathscr {E}, v \in \mathscr {V} \, , \end{aligned}$$

8 $$\begin{aligned}&x_{ij}^v \in \{0,1\} \quad (i,j) \in \mathscr {E}, v \in \mathscr {V} \, , \end{aligned}$$

9 $$\begin{aligned}&f^v_{ij} \ge 0 \quad (i,j) \in \mathscr {E}, v \in \mathscr {V} \, . \end{aligned}$$

The objective function in Eq. (1) is the sum of the fixed vehicle cost for the vehicles used to deliver goods and the total (variable) travel cost for those vehicles. The constraint in Eq. (2) ensures that the maximum number of available vehicles for a specific vehicle type is not exceeded. The constraints in Eqs. (3) and (4) make sure that each customer is visited exactly once, and the constraint in Eq. (5) sees to that all vehicles leaving the depot return to it after delivering their goods. The constraints in Eqs. (6) and (7) ensure a correct commodity flow that meets all customer demands: Eq. (6) specifies that the amount of goods arriving to node *i* minus the amount of goods leaving node *i* should equal the customer demand $q_i$ of customer *i*, and Eq. (7) describes that the amount of goods $f_{ij}^v$ being carried from node *i* to node *j* needs to be at least enough to match customer *j*’s demand $q_j$ and should not exceed the capacity remaining in the vehicle after it delivered its goods to fulfil the demands $q_i$ of customer *i*. Finally, the constraints in Eqs. (8) and (9) enforce the binary form and non-negativity restrictions on the variables.

## Ising formulation for the HVRP

All optimization problems in the complexity class NP can be reformulated as the problem of finding the ground state (lowest-energy configuration) of a Hamiltonian^48^. This is also the method we use for the HVRP in this work. Since the HVRP combines two distinct problems, a routing problem and a capacity problem, we have to derive an Ising Hamiltonian that captures both these problems simultaneously.

### Routing problem

For the routing problem, we start from the TSP formulation in Ref.^48^ with the Hamiltonian $H^\textrm{TSP} = H_A^\textrm{TSP} + H_B^\textrm{TSP}$, where

10 $$\begin{aligned} H_A^\textrm{TSP}&= A \sum _{i=1}^N \left( 1 - \sum _{\alpha =1}^N y_{i \alpha } \right) ^2 + A \sum _{\alpha =1}^N \left( 1 - \sum _{i=1}^N y_{i \alpha } \right) ^2 + A \sum _{(i, j) \notin \mathscr {E}} \sum _{\alpha =1}^N y_{i \alpha } y_{j \alpha +1} \, , \end{aligned}$$

11 $$\begin{aligned} H_B^\textrm{TSP}&= B \sum _{(i, j) \in \mathscr {E}} W_{i j} \sum _{\alpha =1}^N y_{i \alpha } y_{j \alpha +1} \, , \end{aligned}$$

encoding the total cost. Here $N = |\mathscr {N}|$ is the number of nodes including the depot, *A* and *B* are positive constants, and *W* encodes the distances between the nodes. The index *i* represents the nodes and $\alpha$ the order in a prospective cycle. The binary variables $y_{i \alpha }$ can be referred to as ’routing variables’ indicating in which order of the cycle node *i* is visited. There are $N^2$ variables, with $y_{i,N+1}\equiv y_{i,1}$ for all *i*, such that the route ends where it starts. Minimizing the first term in Eq. (10) leads to each node being visited exactly once and minimizing the second term in Eq. (10) ensures that each cycle has exactly one entry for each order. The last term in Eq. (10), which ensures that non-existent arcs are not used, can be neglected for the problem we investigate because we assume a fully connected graph. The Hamiltonian $H_B^\textrm{TSP}$ in Eq. (11) adds up the total distance travelled.

Above, we used $y_{i \alpha }^v$ as a routing variable that tracks the order $\alpha$ in which node *i* is visited within a cycle. To blend this framework with the mathematical structure of the HVRP, formulated in Eqs. (1)–(9), we introduce a transformation from the decision variable *y* to *x* as follows:

12 $$\begin{aligned} x_{ij}^v&= \sum _{\alpha =1}^{N_0-1} y_{i\alpha }^v y_{j\alpha +1}^v \, , \end{aligned}$$

13 $$\begin{aligned} x_{0i}^v&= y_{i1}^v + \sum _{\alpha =2}^{N_0} \left( 1 - \sum _{\begin{array}{c} j=1 \\ j \ne i \end{array}}^{N_0} y_{j \alpha -1}^v \right) y_{i \alpha }^v \, , \qquad x_{i0}^v = y_{i N_0}^v + \sum _{\alpha =1}^{N_0 - 1} y_{i \alpha }^v \left( 1 - \sum _{\begin{array}{c} j=1 \\ j \ne i \end{array}}^{N_0} y_{j \alpha +1}^v \right) \, . \end{aligned}$$

The summation in Eq. (12) is not equal 0 if and only if *i* and *j* are subsequent stops on the same route. Equation (13) ensures that the first and last stops are automatically connected to the depot (assuming a single depot). By using the decision variables $y_{i \alpha }^v$ that indicate the position in a prospective cycle instead of $x_{ij}^v$ that is 1 if and only if a vehicle traverses from customer *i* to *j*^38^, we take care of the subtour-elimination constraint encoded in Eq. (6), which otherwise allows for closed loops between customers, without returning to the depot.

The transformation from $y_{i \alpha }^v$ to $x_{ij}^v$ as defined in Eqs. (12)–(13) is unique. However, the inverse transformation, i.e., from the HVRP model variables $x_{ij}^v$ to the quantum model variables $y_{i \alpha }^v$, is not unique. This becomes evident if we consider that any permutation of the sequence $y_{i \alpha }^v$ for a fixed *i* will yield the same $x_{ij}^v$ values. Hence, a single solution in the HVRP model could correspond to multiple valid solutions in the Ising model. Despite this non-uniqueness, every feasible solution in the HVRP model has a corresponding set of solutions in the Ising model, ensuring the robustness and validity of our approach.

We can now write the Ising formulation for the routing problem. Here we extend the formulation compared to previous works to capture different types of trucks, not just multiple trucks of the same type (having the same capacity)^48,51^. Let $V = |\mathscr {V}|$ be the number of trucks, where $\mathscr {V}$ now is the set of vehicles chosen for the optimization [instead of the set of vehicle *types*, as in Eqs. (1)–(9)], and denote by $N_0 = |\mathscr {N}_0|$ the number of customers to visit. The indices *v* now represent a specific truck of a specific type [instead of just a specific type, as in Eqs. (1)–(9)]. The Ising Hamiltonian $H = H_A + H_B + H_C$ we derive is then merging Eqs. (12)–(13) with Eqs. (1)–(9):

14 $$\begin{aligned} H_{A}&= A \sum _{v=1}^V \sum _{i=1}^{N_0} \sum _{j=1}^{N_0} c^v_{ij} \sum _{\alpha =1}^{N_0 - 1} y_{i \alpha }^v y_{j \alpha + 1}^v + A \sum _{v=1}^V \sum _{i=1}^{N_0} (t_v + c_{0i}^v) \left[ y_{i1}^v + \sum _{\alpha =2}^{N_0} \left( 1 - \sum _{\begin{array}{c} j=1 \\ j \ne i \end{array}}^{N_0} y_{j \alpha -1}^v \right) y_{i \alpha }^v \right] \nonumber \\&\quad + A \sum _{v=1}^V \sum _{i=1}^{N_0} c_{i0}^v \left[ y_{i N_0}^v + \sum _{\alpha =1}^{N_0 - 1} y_{i \alpha }^v \left( 1 - \sum _{\begin{array}{c} j=1 \\ j \ne i \end{array}}^{N_0} y_{j \alpha +1}^v \right) \right] \, , \end{aligned}$$

15 $$\begin{aligned} H_B&= B \sum _{i=1}^{N_0} \left( 1 - \sum _{\alpha =1}^{N_0} \sum _{v=1}^V y_{i \alpha }^v \right) ^2 \, , \end{aligned}$$

16 $$\begin{aligned} H_C&= C \sum _{\alpha =1}^{N_0} \left( 1- \sum _{i=1}^{N_0} \sum _{v=1}^V y_{i\alpha }^v \right) ^2 \, . \end{aligned}$$

The Hamiltonian is composed of different parts. $H_A$ in Eq. (14) captures the total cost of the original mathematical formulation [Eq. (1)], i.e., the minimization of the variable and fixed cost. The first term estimates the variable cost for traveling between the different customers/cities, while the second and third terms measure the cost of leaving and arriving at the depot. Note that the fixed cost is included in the second term as well, since all the vehicles leaving the depot generate the fixed cost.

For this particular mapping it is necessary to define the set of vehicles that are used for the optimization beforehand. Therefore, we can neglect the inequality constraint defined in Eq. (2) from the original formulation, which ensures that the number of vehicles of a specific type does not exceed the number of available vehicles.

The constraint given by $H_B$ in Eq. (15) ensures that each city is visited exactly once [i.e., satisfies the constraints in Eqs. (3) and (4) in the original HVRP formulation]. Furthermore, $H_C$ in Eq. (16) ensures that each city has a unique position in the cycle and that not more than one city can be travelled to at the same time. To make sure that the constraints are not violated, we require $0< \text {max}(|H_A|) < B, C$. If these inequalities are not fulfilled, we risk obtaining unphysical solutions that have lower energies than valid ones.

**Fig. 1:**
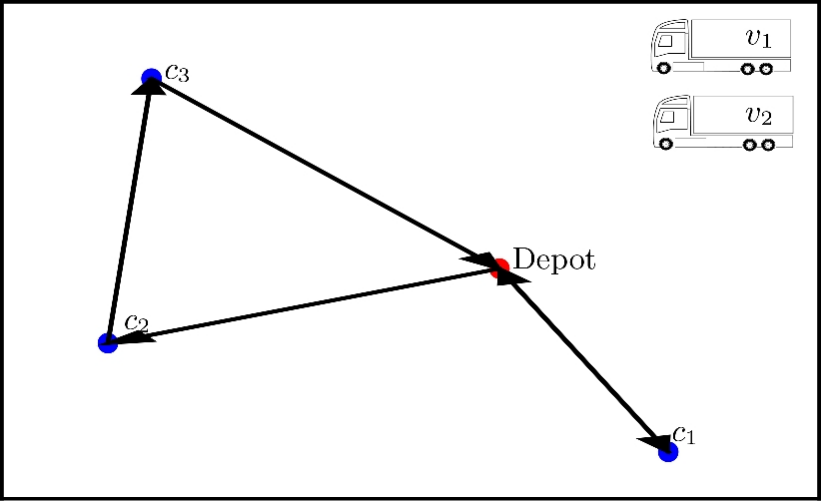
Visualization of a problem instance with a suggested solution (other solutions are possible; we assume a fully connected graph). The first truck $v_1$ visits cities $c_2$ and $c_3$ before returning to the depot. The second truck $v_2$ only visits city $c_1$.

One notable technicality about the formulation is that certain solutions that may be considered valid are excluded by the constraint in Eq. (15). However, the excluded solutions are physically equivalent to some allowed solution, as illustrated by the example in Fig. 1 with two trucks and three cities:

17 $$\begin{aligned} y = \left[ \begin{pmatrix} 0 & 0 & 0 \\ 1 & 0 & 0 \\ 0 & 1 & 0 \end{pmatrix}, \begin{pmatrix} 0 & 0 & 1 \\ 0 & 0 & 0 \\ 0 & 0 & 0 \end{pmatrix} \right] \qquad \text {and} \qquad y = \left[ \begin{pmatrix} 0 & 0 & 0 \\ 1 & 0 & 0 \\ 0 & 1 & 0 \end{pmatrix}, \begin{pmatrix} 0 & 1 & 0 \\ 0 & 0 & 0 \\ 0 & 0 & 0 \end{pmatrix} \right] , \end{aligned}$$

where the matrix rows correspond to the index *i* (the node visited), the matrix columns correspond to the index $\alpha$ (the order in which the nodes are visited), and each matrix corresponds to a vehicle *v*.

In both cases, the two different matrices describe the routes of the two different vehicles and the order in which they visit the different customers $c_i$. They both represent a physically valid solution where the first truck visits first the second and then the third customer ($v_1: c_2 \rightarrow c_3$), while the second truck goes from the depot to the first customer and then back to the depot ($v_2: c_1$). The constraint in Eq. (15), however, rules out the latter representation as it has two non-zero entries for $\alpha =2$. If we want to allow this larger set of viable representations, physically equivalent to solutions already allowed by Eq. (15), we can replace that constraint by a reformulated one,

18 $$\begin{aligned} H'_B = B \sum _{v=1}^V \sum _{\alpha =1}^{N_0} \left( u_\alpha ^v - \sum _{i=1}^{N_0} y_{i \alpha }^v \right) ^2, \end{aligned}$$

where we have introduced $N_0^2$ additional auxiliary qubits $u_{\alpha }^v$. Especially in the NISQ era, where quantum resources are scarce, it is important to encode the problem with as few qubits as possible. Thus we do not consider Eq. (18) a viable route for implementations, but use Eq. (15) for the simulations later in this article.

### Capacity problem

The capacity constraint is of a similar nature as the constraints for the knapsack problem—both are described by an inequality constraint, which for the knapsack problem is to not add too many items to the knapsack and for the trucks to not overload the vehicles. Therefore, we can use the formulation given in Ref.^48^ to model the inequality constraint introduced by the capacities.

The knapsack problem with integer weights is the following. We have a list of *N* objects, labeled by *i*, with the weight of each object given by $w_i$ and its value by $c_i$. The knapsack has a limited capacity of *W*. The binary decision variable $x_i$ denotes whether an item is contained (1) in the knapsack or not (0). The total weight of the knapsack is $\mathscr {W} = \sum _{i=1}^N w_i x_i$ with a total value of $\mathscr {C} = \sum _{i=1}^N c_i x_i$. The knapsack problem is to maximize $\mathscr {C}$ under the constraint $\mathscr {W} \le W$. This problem belongs to the complexity class NP-hard^48^.

We can write an Ising formulation of the knapsack problem as follows. Let $z_n$ for $1 \le n \le W$ be a binary variable which is 1 if the final weight of the knapsack is *n* and 0 otherwise^48^. The Hamiltonian $H^\textrm{K} = H_A^\textrm{K} + H_B^\textrm{K}$ whose energy we seek to minimize is then

19 $$\begin{aligned} H_A^\textrm{K}&= A \left( 1 - \sum _{n=1}^W z_n \right) ^2 + A \left( \sum _{n=1}^W n z_n - \sum _{i=1}^N w_i x_i \right) ^2 \, , \end{aligned}$$

20 $$\begin{aligned} H_B^\textrm{K}&= -B \sum _{i=1}^N c_i x_i \, . \end{aligned}$$

To ensure that the hard constraint is respected, we require $0< \text {max}(|H_B^\textrm{K}|) < A$.

#### Reducing the number of auxiliary qubits

It is possible to reduce the number of variables required for the auxiliary variable $z_n$. We want to encode a variable which can take the values from 0 to *W*. Let $M \equiv \lfloor \text {log}_2(W) \rfloor$. We then require $M+1$ binary variables instead of *W* binary variables:

21 $$\begin{aligned} \sum _{n=1}^W n z_n \rightarrow \sum _{m = 0}^{M-1} 2^{m} z_m + \left( W + 1 - 2^M \right) z_M. \end{aligned}$$

It is important to note that in this encoding method, multiple bitstrings $z_m$ can represent the same number. This situation arises due to the transition from one-hot encoding to a binary representation. In one-hot encoding, each integer from 0 to *W* would have a unique bitstring representation. However, in our binary representation, where we encode numbers using fewer bits, different bitstrings can be interpreted as the same integer. This degeneracy is possible if $W \ne 2^{M+1} -1$.

We can make use of the inequality constraint given in the knapsack formulation [Eq. (19)] to encode the capacity constraints for the HVRP. Therefore, we can neglect $H_B^\textrm{K}$ [Eq. (20)] and only consider $H_A^\textrm{K}$ [Eq. (19)]. Let $Q^v$ be the maximum capacity of vehicle *v*. The Hamiltonian then becomes

22 $$\begin{aligned} H^\textrm{K}&= A \sum _v \left( 1 - \sum _{k=0}^{Q^v} z_k^v \right) ^2 + A \sum _v \left( \sum _{k=0}^{Q^v} k z_k^v - \sum _{\alpha ,i} q_i y_{i \alpha }^v \right) ^2, \end{aligned}$$

or equivalently, using the log formulation,

23 $$\begin{aligned} H^\textrm{K}&= A \sum _v \left( \sum _{k=0}^{M^v-1} 2^k z_k^v + (Q^v + 1 - 2^{M^v}) z_{M^v}^v - \sum _{\alpha ,i} q_i y_{i \alpha }^v \right) ^2, \end{aligned}$$

where $M^v \equiv \lfloor \text {log}_2(Q^v) \rfloor$. Note that by using the log trick, the decision variable $z_k^v$ switches from a one-hot encoding to a binary representation. The lowest energy possible for the Hamiltonian in Eq. (23) is zero; this minimum is reached when the $z_k^v$ are chosen such that each vechicle *v* carries an amount of goods, given by the first inner sum in Eq. (23), not exceeding its maximum capacity, and that amount exactly corresponds to the demand of the customers it visits along its route, given by the second inner sum in Eq. (23).

### The full Ising Hamiltonian for the HVRP

We are now ready to write down the full Hamiltonian for the HVRP. The full Ising Hamiltonian $H_{\mathscr {C}}$ contains four terms, where the first term $H_A$ captures the actual optimization problem and the other terms are penalty terms to ensure that invalid configurations are penalized with a high energy:

24 $$\begin{aligned} H_{\mathscr {C}}&= H_A + H_B + H_C + H_D \, , \end{aligned}$$

25 $$\begin{aligned}&\begin{aligned} H_D&= D \sum _{v=1}^V \left( \sum _{k=0}^{M^v-1} 2^k z_k^v + (Q^v + 1 - 2^{M^v}) z_{M^v}^v - \sum _{\alpha =1}^{N_0} \sum _{i=1}^{N_0} q_i y_{i \alpha }^v \right) ^2 \end{aligned} \end{aligned}$$

For the terms $H_A$ to $H_C$, see Eqs. (14)–(16). Note that $H_D$, together with $H_B$, ensures that we satisfy the commodity-flow constraints in Eqs. (6) and (7) in the original HVRP formulation.

The formulation presented in this paper combines the capacity problem and the routing problem in one Ising Hamiltonian. Similarly, a unified approach is also attempted in Ref.^31^ with the difference that the authors add a constraint for clustering the customers as well. Here, by using the decision variables $y_{i \alpha }^v$ that indicate the position in a prospective cycle instead of $x^v$ that is 1 if and only if a vehicle traverses from customer *i* to *j*, we circumvent the subtour-elimination constraint. This constraint needs to loop through all possible subtours as it is presented in Ref.^38^. Moreover, a solution obtained with our mapping does not necessarily use all the vehicles that are available. It can find the most cost-efficient subset of vehicles needed to solve the task.

### Resources

The required resources (qubits) for solving the HVRP with our approach can be separated into two parts. The first part comes from representing all the possible routes between the customers in the routing part of the problem and scales with $N_0^2 \cdot V$. Additional qubits are required for the constraining term $H_D$. The total number of qubits required is

26 $$\begin{aligned} \#q = N_0^2 \cdot V + \underbrace{\sum _{v=1}^{V} \lfloor \log _2 Q^v \rfloor + 1}_{H_D}. \end{aligned}$$

Using the alternative formulation for $H_B$ [see Eq. (18)] adds more auxiliary qubits ($N_0 \cdot V$), yielding

27 $$\begin{aligned} \#q =(N_0 + 1) N_0 V + \sum _{v=1}^{V} \lfloor \log _2 Q^v \rfloor + 1. \end{aligned}$$

Furthermore, we can estimate the number of single- and two-qubit gates required to apply the QAOA (see next section) to the problem in this formulation. For the routing part of the problem, each layer of the QAOA needs $O (N_0^2 \cdot V)$ single-qubit gates (i.e., proportional to the number of qubits) and $O (N_0^3 \cdot V)$ two-qubit gates (i.e., proportional to $N_0$ times the number of qubits). For the capacity part of the problem, the number of single-qubit gates is the same, while the number of two-qubit gates is $O (N_0^2 \cdot Q)$. What this translates to in circuit depth will depend on the connectivity of the quantum hardware.

As a comparison, we note that modern high-performance optimizers (classical computers) for the HVRP can find near-optimal solutions for problem instances with more than 1,000 customers^52,53^. Typically, these optimizers exhibit polynomial or pseudo-polynomial time complexity. For a quantum computer to solve problem instances of this size, it would need at least millions of controllable qubits. Systems of this size are likely still several years away from being realized.

## The quantum approximate optimization algorithm

**Fig. 2:**
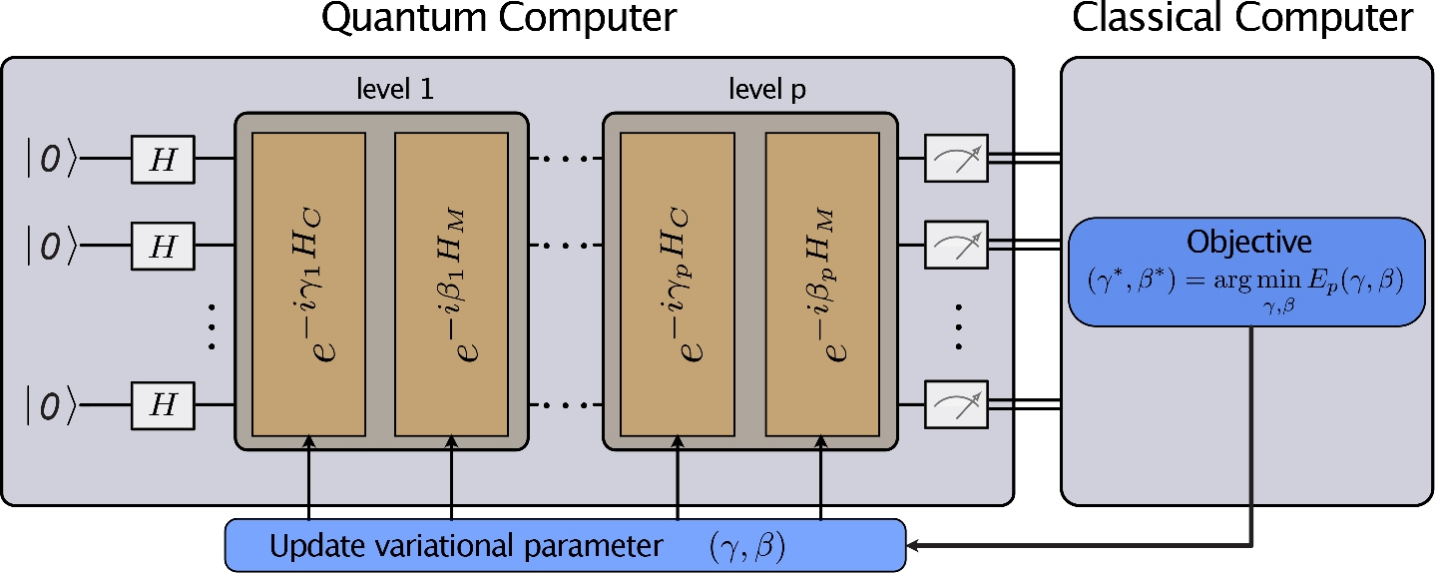
A schematic description of the QAOA, visualizing the interplay between the quantum device and the classical computer. The quantum computer implements a variational state formed by applying *p* parameterized layers of operations. Each layer has operations involving the cost Hamiltonian $H_C$ and a mixing Hamiltonian $H_M$, weighted by the angles $\gamma$ and $\beta$, respectively. Measurements of the variational state and calculations of its resulting energy are used to guide the classical optimizer, which minimizes the energy in a closed-loop optimization.

The QAOA belongs to the class of hybrid quantum-classical algorithms, which combine quantum and classical processing. The closed-loop optimization of the classical and quantum devices is visualized in Fig. 2. The quantum subroutine, operating on *n* qubits, consists of a consecutive application of two non-commuting operators defined as^9^

28 $$\begin{aligned} U(\gamma )&\equiv e ^{-i\gamma H_\mathscr {C}} \quad \gamma \in [0, 2\pi ] \, , \end{aligned}$$

29 $$\begin{aligned} U(\beta )&\equiv e^{-i\beta H_M} = \prod _{j=1}^n e^{-i\beta \sigma ^x_j} \quad \beta \in [0, \pi ] \, , \end{aligned}$$

where $\sigma ^x$ denotes the Pauli *X* matrix. This operation is analogous to the classical NOT gate. It changes the $\left| 0\right\rangle$ state to the $\left| 1\right\rangle$ state, and vice versa. The operator $U(\gamma )$ gives a phase rotation to each bit string depending on the cost of the string, while the mixing term $U(\beta )$ scrambles the bit strings. We call $H_\mathscr {C}$ the cost Hamiltonian and $H_M$ the mixing Hamiltonian [Eq. (29) is a transverse-field mixer]. The bounds for $\gamma$ and $\beta$ are valid if $H_\mathscr {C}$ has integer eigenvalues^9^. The formulation of $H_\mathscr {C}$ for the HVRP is given by Eq. (24).

The initial state for the algorithm is a superposition of all possible computational basis states. This superposition can be obtained, as shown in Fig. 2, by first preparing the system in the initial state $\left| 0\right\rangle ^{\otimes n}=\left| 00 \ldots 0\right\rangle$ for all qubits and then applying the Hadamard gate on each qubit:

30 $$\begin{aligned} \left( \tilde{H}\left| 0\right\rangle \right) ^{\otimes n}&= \left( \frac{\left| 0\right\rangle + \left| 1\right\rangle }{\sqrt{2}}\right) ^{\otimes n} \equiv \left| +\right\rangle ^{\otimes n} \, , \end{aligned}$$

where $\otimes$ denotes the tensor product and $\tilde{H}$ the Hadamard gate.

For any integer $p \ge 1$ and 2*p* angles $\gamma _1 \dots \gamma _p \equiv \varvec{\gamma }$ and $\beta _1 \dots \beta _p \equiv \varvec{\beta }$, we define the angle-dependent quantum state

31 $$\begin{aligned} \left| \varvec{\gamma , \beta }\right\rangle = U( \beta _p) U(\gamma _p) \dots U(\beta _1) U( \gamma _1) \left| +\right\rangle ^{\otimes n}. \end{aligned}$$

The quantum circuit parametrized by $\varvec{\gamma }$ and $\varvec{\beta }$ is then optimized in a closed loop using a classical optimizer. The objective is to minimize the expectation value of the cost Hamiltonian $H_{\mathscr {C}}$^9^, i.e.,

32 $$\begin{aligned} (\varvec{\gamma ^*, \beta ^*}) =\underset{\varvec{\gamma , \beta }}{\text {argmin }} E(\varvec{\gamma , \beta }), \qquad E(\varvec{\gamma , \beta }) = \left\langle \varvec{\gamma , \beta }\right| H_{\mathscr {C}} \left| \varvec{\gamma , \beta }\right\rangle . \end{aligned}$$

The problem of calculating the energy of $2^{\#q}$ possible bit strings (solutions) is thus reduced to a variational optimization over 2*p* parameters.

The integer *p* plays a crucial role in the overall solution quality. The performance of the algorithm is expected to become better with more variational parameters. Furthermore, there exists a close connection between QAOA and adiabatic quantum computing: the QAOA can reproduce a Trotterized version of the time evolution in adiabatic quantum computing^9,54^.

## Benchmarking quantum approximate optimization for the heterogeneous vehicle routing problem

**Table 1:** Information about the three different problem instances used in simulations.

Problem instance	I	II	III
Number of cities	3	4	3
Number of trucks	1	1	2
Number of qubits for routing	9	16	18
Number of qubits for capacities	2	3	3
Total number of qubits	11	19	21

**Fig. 3:**
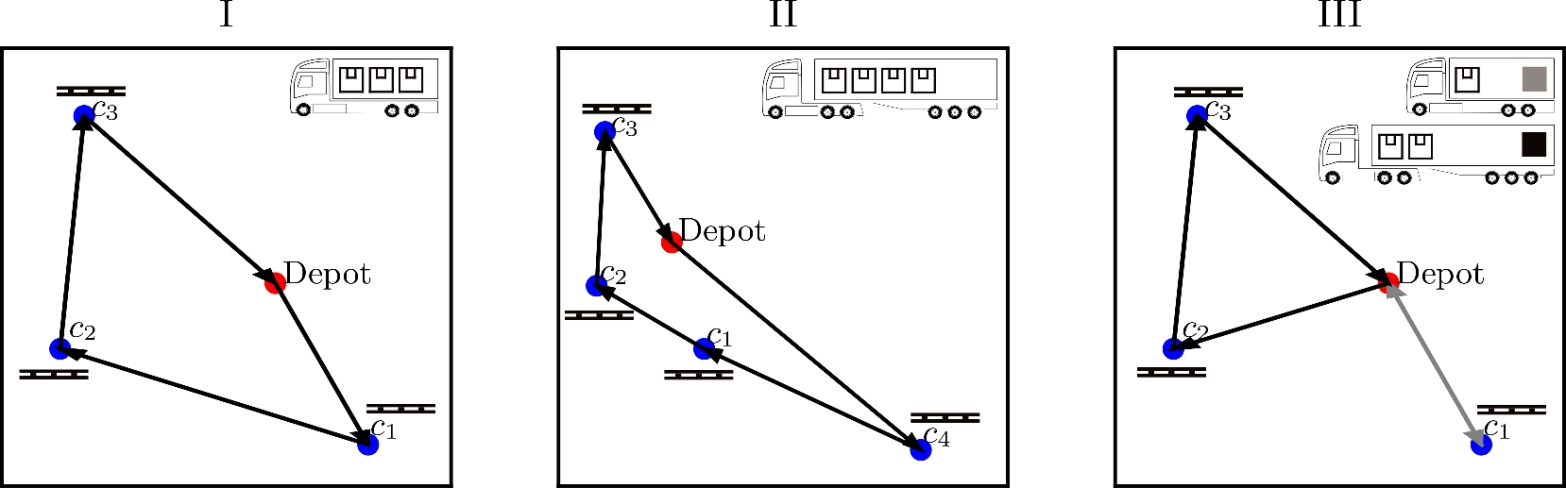
A visual representation, with correct scaling, of the three problem instances that are considered for the simulations. The optimal solution is shown. Each truck carries a predefined amount of goods and brings it to the respective customers. The pallets indicate that one item has to be carried to the customer. The crates show how much goods is carried by each truck. A color coding indicates the route assignment for problem instance III. The optimal order in which the customers are visited is indicated by arrows (the reverse order is also optimal).

In this section, we show numerical results from noise-free simulations (exact state-vector simulations implemented in Python, without decoherence during the time evolution and without shot noise [finite-sampling effects] in the measurement results) of the QAOA applied to the HVRP. We examine three different problem instances, labelled I, II, and III, which use 11, 19, and 21 qubits, respectively. For these problem sizes, we can obtain the optimal solution from a classical brute-force search and compare it with the solutions obtained from the QAOA. Table 1 contains information about the number of cities, available trucks, and the overall number of qubits needed to encode these problem instances in an Ising Hamiltonian using the scheme we have described above. For the simulations, we consider realistic fuel consumption, gas prices, and fixed costs for each truck type, as detailed in the Supplementary Information. A graphical representation of the problem instances is shown in Fig. 3.

For the simulations we consider two different approaches. One is to only solve for satisfying the constraints. The other is to solve the full problem, optimizing not only for feasible solutions, but for the best solution. This stepwise approach, starting with the constraints [Eqs. (15), (16), and (25)] and then including the optimization part [(Eq. (14)], helps us understand whether some parts of the full problem contribute more to its difficulty than others.

For the first approach, finding satisfying solutions, we neglect the actual optimization part of the problem, i.e., minimizing the cost of the routing. We set the prefactors of the different parts of the Hamiltonian [Eqs. (15), (16), and (25)] to 1. The eigenvalues of the Hamiltonian are integers. This allows us to restrict the search space for $\beta$ and $\gamma$ in Eqs. (28)–(29) to $[0, \pi ]$ and $[ 0, 2 \pi ]$, respectively. For the full problem, we cannot make use of this simplification.

The second approach is to solve the full problem with the optimization (cost minimization) included [Eqs. (14)–(16) and Eq. (25)]. We rescale the cost function of the optimization [Eq. (14)] such that it only takes values between 0 and 1. Note that for this rescaling, we have to find the highest cost allowed by the cost function, which requires evaluating the cost for each possible solution in a brute-force approach. This is only feasible for small problem instances. In a real-world application, this rescaling procedure thus cannot be applied (although it may be possible to carry it out by estimating the highest possible cost). It may then be necessary to optimize over the penalty weights *A*, *B*, *C*,  and *D* as well^51,55^. The eigenvalues of the full Ising Hamiltonian [Eq. (24)] are then no longer integers and thus we cannot set bounds for the variational parameters (in our simulations below, we initialize $\gamma$ and $\beta$ in the range $[0, 2\pi ]$, but after optimization they may end up with values outside that range).

For real-world applications, a small probability for finding the optimal bitstring can still be sufficient, since we can efficiently evaluate the cost of the bitstring with a classical computer.

### Energy landscape

**Fig. 4:**
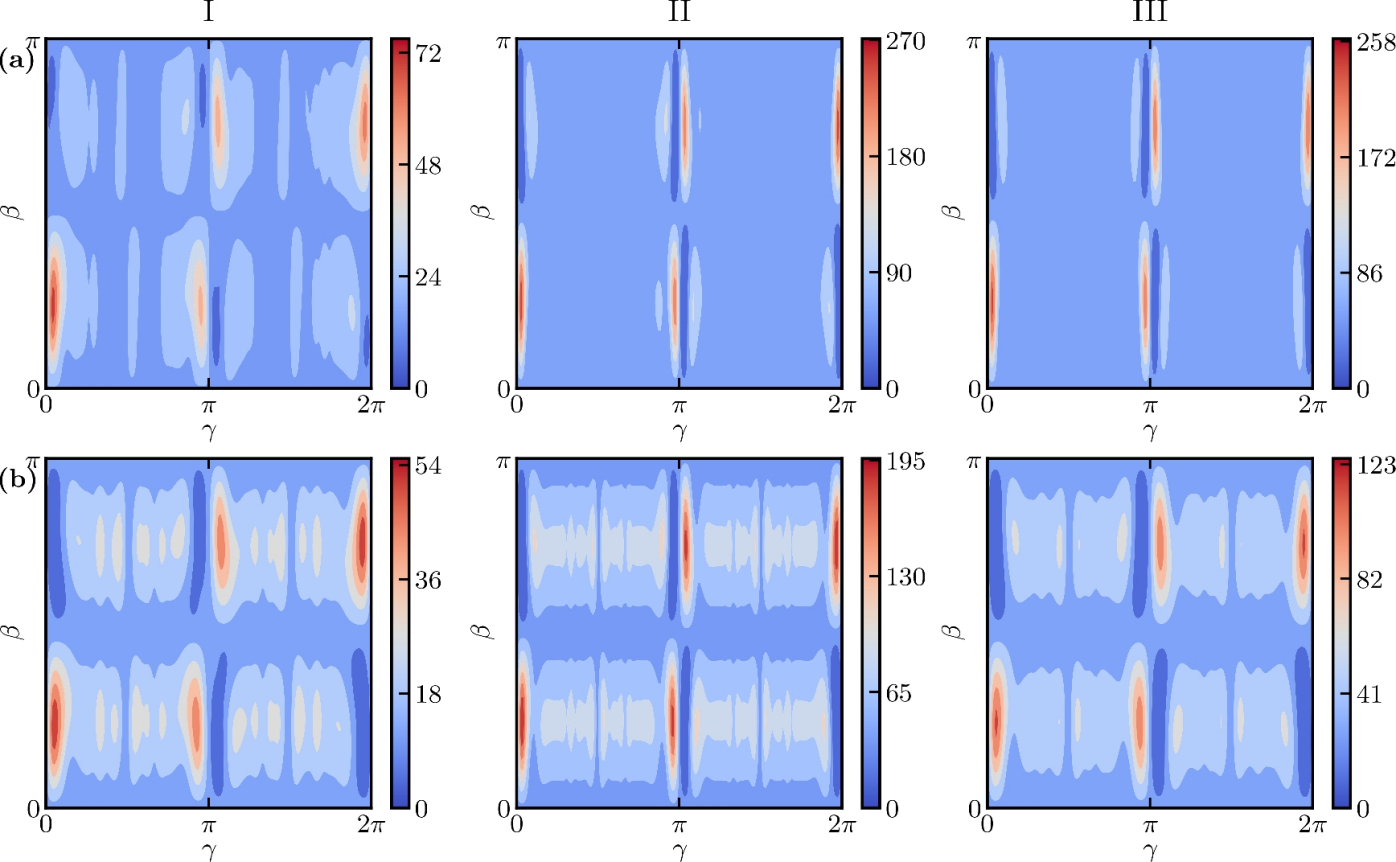
Energy landscapes for the three problem instances with circuit depth $p=1$. (**a**) The energy landscape for the full HVRP [Eqs. (14)–(16) and Eq. (25)]. The expectation value $E(\gamma , \beta )$ for the total cost depends on the classically optimized angles $\gamma$ and $\beta$. The periodicity is broken due to the non-integer eigenvalues for the cost Hamiltonian. (**b**) The energy landscape for the capacity constraint only [Eq. (25)]. Here, the expectation value $E(\gamma , \beta )$ describes the energy penalty for breaking the capacity constraint.

To illustrate the difficulty of finding good variational parameters for the QAOA, we show in Fig. 4 the energy landscape for $p=1$ for each problem instance, considering the full problem [Fig. 4a] and the capacity constraint in isolation [Fig. 4b]. We evaluate the expectation value $E (\gamma , \beta )$ of the cost Hamiltonian on a grid $\{ \gamma , \beta \} \in [0, 2 \pi ] \times [ 0, \pi ]$. Note that for the full problem, no such bounds can be set for the variational parameters, but for this visualization we constrain it to the range mentioned.

The states with the lowest energy are marked with dark blue in Fig. 4. Each surface plot shows four distinct optima. Moreover, the variational parameters are concentrated in the same region for all three problem instances, both for the full problem and for only the capacity constraint. The range for optimal parameters narrows with increasing problem size. Similar behaviour has been observed in several other studies, e.g., in Refs.^56,57^. The overall shape of the energy landscape does not change significantly with varying problem size, but the overall expectation value increases. This is not surprising, since with increasing problem size there are many more constraints to satisfy, goods to deliver, and trucks to choose from.

As discussed earlier in this article, the HVRP consists of two problems, a routing problem and a capacity problem. The latter is analogous to the constraints of the knapsack problem^48^. To better understand the energy landscapes for the capacity part of the problem instances shown in Fig. 4b, we now take a closer look at the landscape of a particular knapsack problem. The problem is: given a knapsack with a maximum capacity of 5, choose from the list of items [4, 3, 2, 1] the ones that satisfy the capacity constraint. Seven qubits are needed to encode the problem, making use of the log trick introduced in Eq. (21).

**Fig. 5:**
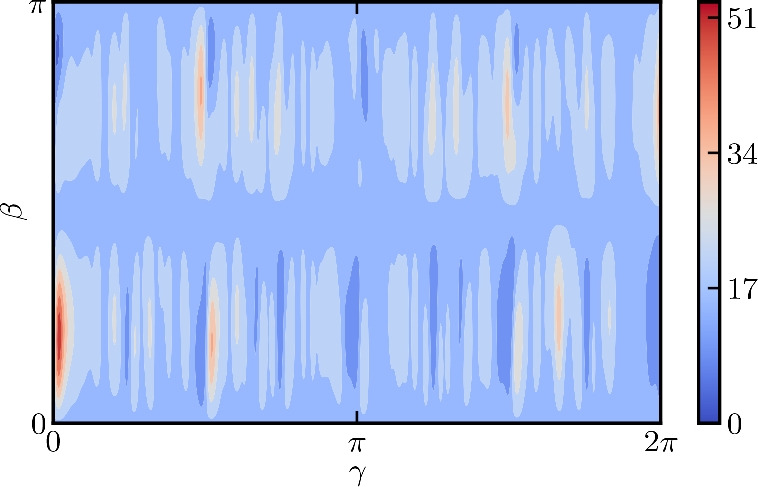
The energy landscape for the knapsack problem [see Eq. (19)] discussed in the main text with a circuit depth of $p=1$. The energy landscape is highly non-convex and finding the global minimum quickly can be difficult for a classical optimizer.

Figure 5 shows the (rapidly oscillating) energy landscape for this knapsack problem. It is clear that many optimizers may struggle to find the global optimum quickly in this landscape. We argue that with increasing complexity of the problem instances for the HVRP, maneuvering the landscape of the capacity constraint becomes a difficult problem. In Fig. 4b, we do not observe this behaviour yet, but this is simply due to the fact that the problem instances we consider are small (see Table 1). To obtain a landscape that is easier to handle for the classical optimizer, it might be necessary to relax the knapsack constraint or to find a different formulation to encode the capacity constraint^58^.

### Increasing the circuit depth

**Fig. 6:**
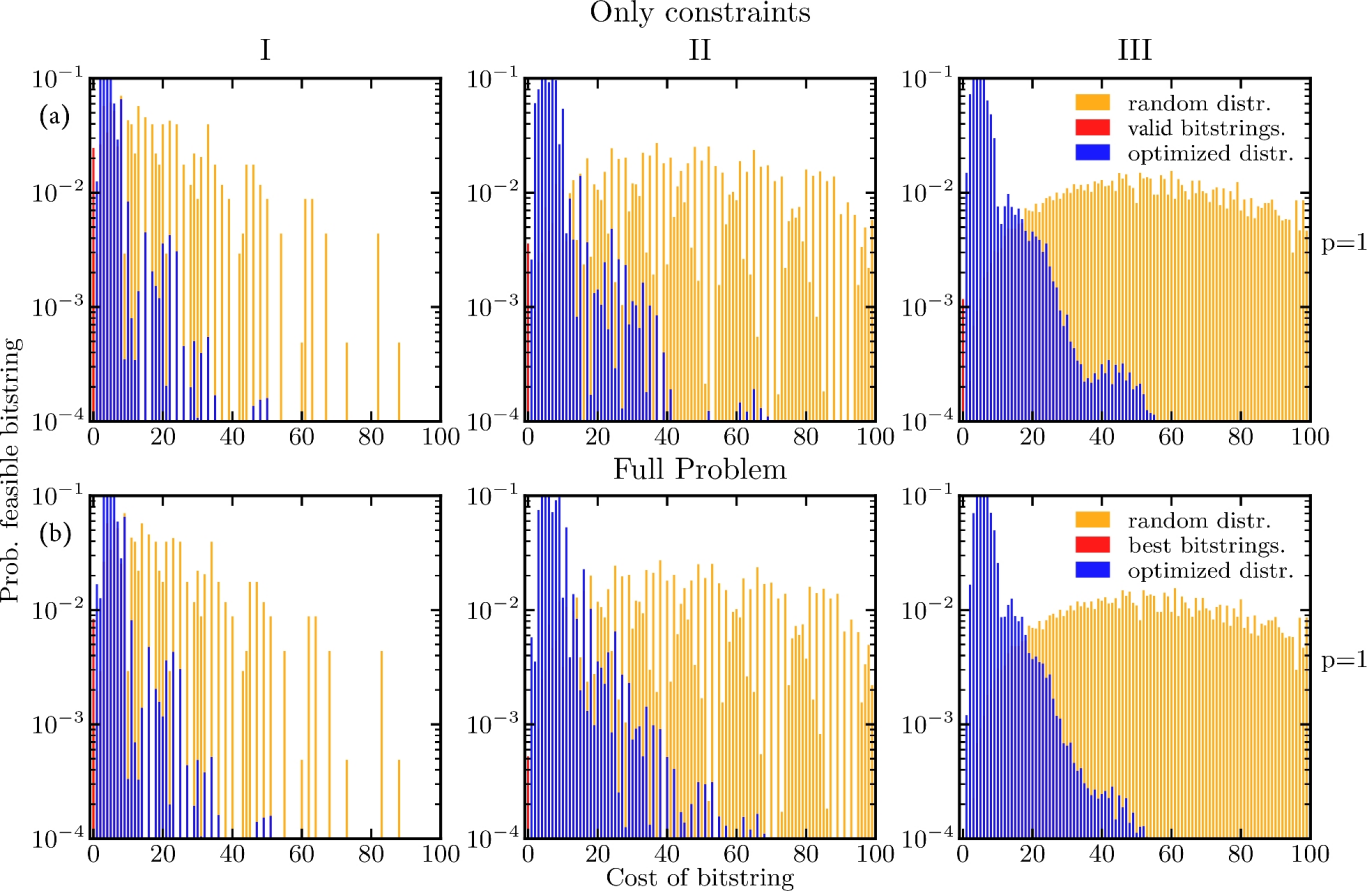
(**a**) The probability distribution of the optimized variational state (red, blue) for $p=1$; the goal of the optimization is to minimize the cost. The color red shows the probability for finding a valid solution to the routing problem, i.e., a solution satisfying all constraints. The blue color indicates the overall outline of the optimized probability distribution. As a comparison, we show the probability distribution for the $\left| +\right\rangle ^{\otimes n}$ state, meaning all bitstrings are sampled with uniform probability (orange). (**b**) The probability distribution of the optimized variational state considering the full problem (red, blue) for $p=1$. The probability distribution is binned such that *i* is the largest possible integer where $i \le x$ holds, also denoted as $\lfloor x \rfloor$. That results in all feasible solutions being binned to zero and all the others being binned to integers indicating the number of constraint violations.

It has been shown that for a circuit depth of $p=1$, the QAOA cannot outperform classical optimization algorithms^9,59^. For actual applications of the QAOA, it is therefore necessary to go to a circuit depth beyond $p=1$.

Before we investigate $p>1$, we start with the lowest possible circuit depth of $p=1$. In Fig. 6, we show histograms of the probability distributions created by the variational circuit for finding solutions satisfying all constraints, in the cases where the cost function encodes only the constraints and the full problem. We show the probability of sampling bitstrings with a specific cost (there can be several bitstrings leading to a particular cost). The probability of sampling any of the feasible bitstrings is shown in red and the rest of the optimized distribution is depicted in blue. As a comparison, we show the probability distribution for the $\left| +\right\rangle ^{\otimes n}$ state, meaning all bitstrings are sampled with uniform probability (orange). The difference between the distributions in Fig. 6a and Fig. 6b is marginal. Thus, adding the cost term [Eq. (14)] to the optimization problem does not alter the overall performance of the algorithm when it comes to satisfying the constraints. This is perhaps not so surprising when considering that due to the rescaling of the cost Hamiltonian, the costs not associated with constraints impact the overall shape of the energy landscape less.

The optimized probability distribution is shifted to the left compared to the random distribution. Thus, sampling from the optimized variational state gives solutions with overall lower energy compared to random sampling of bitstrings. Moreover, for the small problem instance with 11 qubits, it is possible to sample valid bitstrings with a probability of approximately 3 %. For the 19- and 21-qubit problem instances, the probability of sampling a valid bitstring is about an order of magnitude less. This is not surprising since we are limiting the algorithm to a shallow circuit depth.

**Fig. 7:**
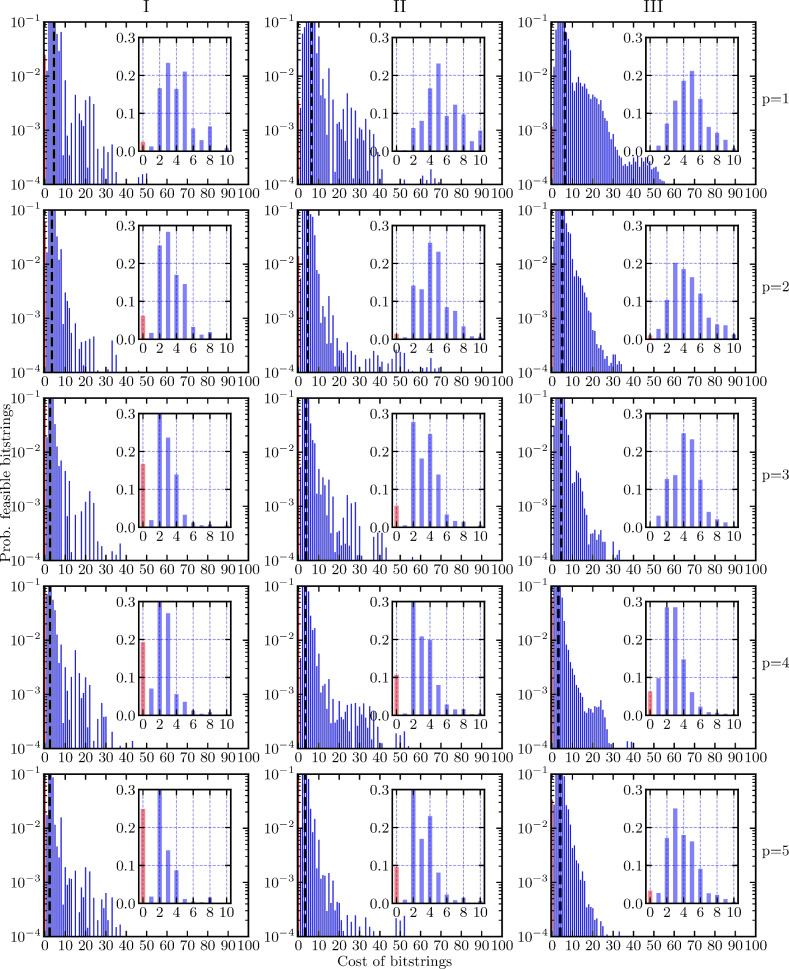
The probability distribution of the optimized variational state $\left| \varvec{\gamma , \beta }\right\rangle$ for the 11- (left), 19- (middle) and 21-qubit (right) problem instances as a function of the circuit depth *p* for obtaining feasible tours. The circuit depth increases from top $p=1$ to bottom $p=5$, leading to a shift towards lower-energy eigenstates. The average energy of the variational state is marked by a dashed black line in each panel. The probability of sampling bitstrings that encode the optimal (lowest-energy) solution is marked with red. The simulations were conducted with the classical optimizer basinhopping with the local optimizer Broyden–Fletcher–Goldfarb–Shanno (BFGS) (see below).

**Fig. 8:**
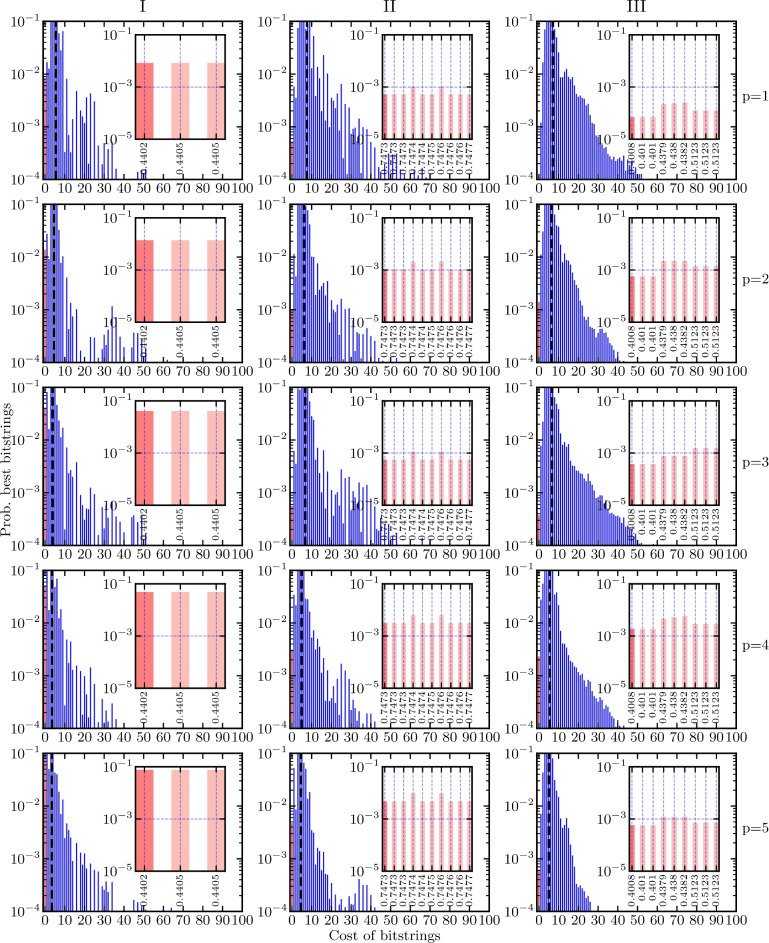
The probability distribution of the optimized variational state $\left| \varvec{\gamma , \beta }\right\rangle$ for the 11- (left), 19- (middle), and 21-qubit (right) problem instances as a function of the circuit depth *p* for finding the best tour. The circuit depth increases from top $p=1$ to bottom $p=5$, leading to a shift towards lower-energy eigenstates. The average energy of the variational state is marked by a dashed black line in each panel. The probability of sampling the best (lowest-energy) bitstrings is marked with red. The binning is done in the same way as in Fig. 6b. The inset shows the probability for sampling any of the two best bitstrings (dark red, leftmost bin) and the probabilities for sampling any of the other feasible bitstrings (light red). The simulations were conducted with the classical optimizer basinhopping with the local optimizer BFGS (see below).

Next, we consider $p \ge 1$. In Figs. 7 and 8, we look at feasible solutions and the best solutions, respectively. We show how the variational state changes with increasing circuit depth from $p=1$ to $p=5$ for the three problem instances [see Fig. 3]. Additionally, we show for Fig. 7 an inset focusing on the low-energy part of the distribution of the variational state and for Fig. 8 an inset focusing on all feasible bitstrings. Again, the bitstrings we are aiming to sample are marked in red and the rest of the optimized distribution is depicted in blue.

For the 11-qubit instance, the probability of sampling a valid bitstring reaches values up to 25 % for $p=5$. For the slightly larger instances (19 and 21 qubits), the probability of sampling a valid bitstring does not exceed 10 % (see Fig. 7). For the more difficult problem of actually finding the optimal tour (not just a feasible tour), the probability drops to 10 % for the 11-qubit instance, while for the 19- and 21-qubit instances the probability of sampling the ideal bitstring is around 1 % or below (see Fig. 8).

The inset in Fig. 8 shows that the algorithm cannot distinguish between the different feasible solutions and thus fails to optimize for the best solution. This might be due to the very small energy gap between the lowest and the first excited energy eigenstate, which is an artifact of the rescaling of the cost, compared to the energy penalty for violating constraints, that we introduced earlier. The point of this rescaling is to ensure that all feasible solutions have lower energy than any solution violating any constraint. Rigorous hyperparameter optimization might be necessary to weight the cost and the constraints to circumvent the problems introduced by the chosen rescaling^51^.

The probability of sampling bitstrings with low cost increases with increasing circuit depth, meaning that the probability distribution shifts to lower-energy eigenstates. This trend is visible in Figs. 7 and 8 as the probability distribution and the average energy (marked by the dashed black line in each panel) of the optimized state shifts increasingly to the left with increasing depth. It may be possible with increasing circuit depth to obtain higher probabilities of sampling optimal solutions at the expense of optimizing more variational parameters. This is in accordance with the theory of QAOA—the performance of the algorithm improves with more variational parameters (increasing depth). The drawback is that the optimization problem becomes increasingly difficult and time-consuming. It is therefore important to select a good optimization method.

### Performance comparison of classical optimizers

Optimizers for finding the variational parameters play an important role in the context of variational quantum algorithms (VQAs). Much of the current research aims to find optimizers that perform well on such quantum circuits^60–63^ and there is a rich literature proposing different optimizers for different problems^3,64^. In the following, we analyze the performance of simulations using four different well-known optimizers: Nelder–Mead^65^, Powell^66^, differential evolution^67^, and basinhopping^68^. The selected optimizers work differently: some use global search mechanisms, e.g., multiple random initial guesses (100 were used for differential evolution), while others use only a single random initial guess as a starting point for the optimization. These characteristics are crucial to understand why the performance of the optimizer varies.

The Nelder–Mead method uses a geometrical shape called a simplex to search the function space. With each step of the optimization, the simplex shifts, ideally, towards the region with a minimum. The Nelder–Mead algorithm belongs to the class of gradient-free optimizers. The Powell optimizer works for non-differentiable functions; no derivatives are needed for the optimization. The method minimises the function by a bi-directional search along each search vector. The initial search vectors are typically the normals aligned to each axis. The differential evolution algorithm is stochastic in nature and does not rely on derivatives to find the minimum. This algorithm often requires larger numbers of function evaluations than conventional gradient-based techniques. The basinhopping optimization algorithm is a two-phase method, which couples a global search algorithm with a local minimization at each step. For the simulations here (including in the preceding sections), we used the Broyden–Fletcher–Goldfarb–Shanno (BFGS) algorithm^69^ as a local optimizer. This algorithm approximates the descent direction by gradually improving the approximation of the Hessian matrix of the loss function determined by the (approximated) gradient. This framework has been proven useful for hard nonlinear optimization problems with multiple variables^70^.

To speed up the optimization routines, we use the optimized parameters from $p-1$ as an initial guess for the optimization of the variational circuits for $p>1$, as well as the optimized parameters from the 11-qubit problem instance as an initial guess for the 19- and 21-qubit instances. The concentration of variational parameters for the QAOA has been observed by many researchers^56,57^. It has been shown for a circuit depth of $p=1,2$ that the variational parameters for small problem instances can be used to infer parameters for larger problem instances^57^. This can significantly speed up the optimization of the QAOA.

**Fig. 9:**
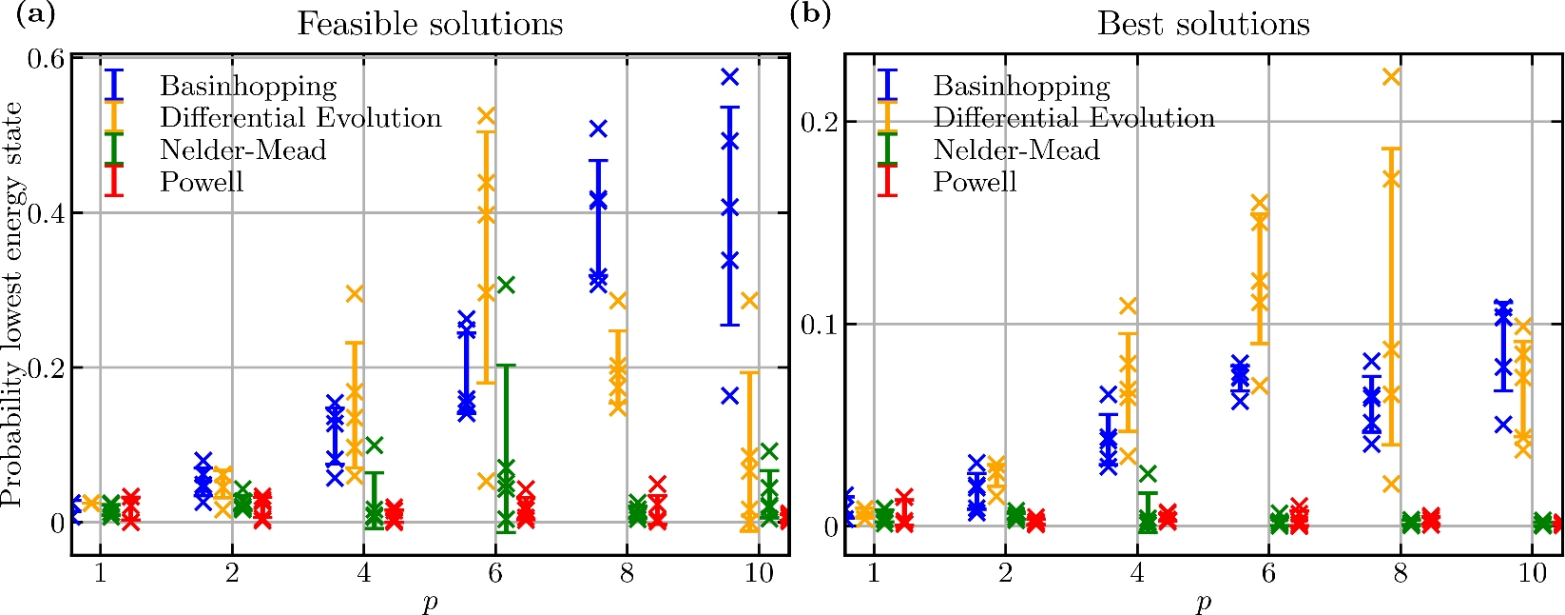
Comparing different optimizers for the HVRP on the 11-qubit problem instance shown in Fig. 3. A probability of 1 means that a feasible bitstring is always sampled. (a) Success probability for finding a valid solution with increasing circuit depth *p*. (b) Success probability for finding the best solution with increasing circuit depth *p*. For each optimizer and each *p*, 10 runs are performed, each from a random initialization and each result marked with a cross. The error bars indicate ± one standard deviation around the mean of the 10 runs. The Nelder–Mead and Powell optimizers run until convergence. The differential evolution and basinhopping optimizers are capped at 1,000 and 50 iterations, respectively; their global search methods tend not to converge.

In Fig. 9a, we show the success probability of finding a viable bitstring (giving the lowest energy for the Hamiltonian specifying the constraints) as a function of the circuit depth *p* for the 11-qubit problem instance. Similarly, we show the success probability of sampling one of the two optimal tours (giving the lowest energy for the full Hamiltonian) as a function of the circuit depth *p* in Fig. 9b. One of the optimal tours is shown in Fig. 3. The other optimal solution is following the same path in reverse order.

The results in Fig. 9 clearly favour the optimizers basinhopping and differential evolution. They perform significantly better than the Nelder–Mead and Powell optimization algorithms. Indeed, the Nelder–Mead and Powell algorithms fail to improve the success probability when *p* increases, which suggests they are not suitable for QAOA. This difference in performance might be due to the global optimization routine that these latter two algorithms use to find the optima. The performance of the Nelder–Mead algorithm strongly depends on the initial simplex and the simplex is usually randomly generated. Depending on the starting point, the performance can vary, but it usually cannot compete with the solution quality of the basinhopping or differential-evolution algorithms.

### Runtime comparison of the classical optimizers

**Fig. 10:**
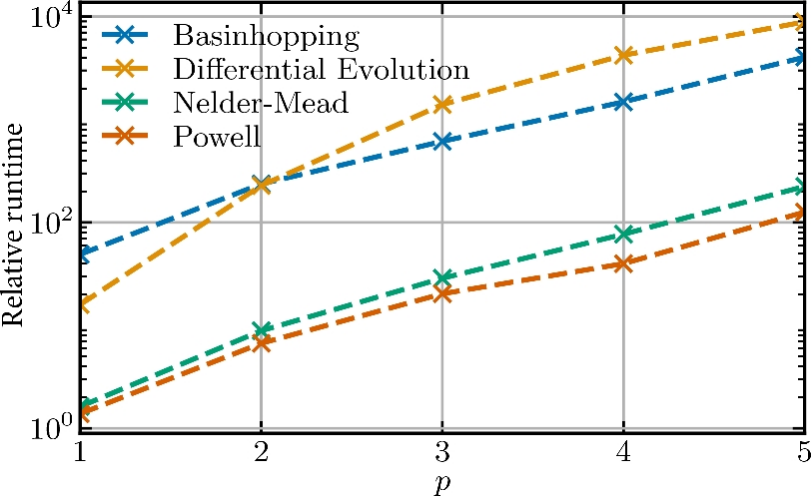
Runtime comparison for the four classical optimizers tested in this work. The plot shows the wall-clock time for a full optimization run as a function of circuit depth *p* for the 11-qubit instance. A time unit, corresponding to a single optimization step of the Powell algorithm with $p=1$, is 10 seconds.

For applications it is important to understand the scaling of the runtime of the optimization routine with increasing circuit depth. Here we benchmark the classical optimizers from the preceding subsection on this measure. The result is shown in Fig. 10.

We see that the optimizers basinhopping and differential evolution need roughly one order of magnitude more time for the optimization than the Nelder–Mead and Powell algorithms. This is due to the many circuit queries the former optimizers have to perform. The tradeoff between algorithm runtime and performance becomes evident, as the slowest optimizers in terms of runtime performed best in terms of success probability (see Fig. 9). Moreover, the linear increase for all optimizers on the semi-log scale in Fig. 10 indicates that the amount of time needed for the optimization scales exponentially with *p*. However, further investigation is needed to confirm this statement. It might become a difficult problem to tackle when a circuit depth beyond $p=20$ is considered.

As mentioned earlier, researchers are already investigating how the optimization could be simplified or circumvented completely^56,57^. The results here further underline the importance of such research.

## Conclusion

We have derived an Ising formulation for the heterogeneous vehicle routing problem (HVRP) under consideration of all relevant constraints, enabling this problem to be solved on a quantum computer or a quantum annealer. In our formulation, the number of qubits needed to encode a problem instance scales quadratically with the number of customers. Therefore, quantum computers will need to have at least millions of qubits to use our suggested encoding scheme to solve problem instances that are at the limit of what today’s classical high-performance optimizers can solve. A quantum advantage could still be had with fewer qubits for smaller problems if they could be solved faster on a quantum computer than on a classical one, but the present work did not give indications of such speed-ups for small problems.

We simulated solving small instances of the HVRP and with the quantum approximate optimization algorithm (QAOA). We considered three distinct problems, requiring 11, 19, and 21 qubits, respectively. We investigated the performance of the algorithm with respect to two design choices: the classical optimizer and the depth *p* of the quantum circuit.

For the choice of optimizer, we found that the basinhopping and differential-evolution algorithms seem well suited to optimize the variational parameters of the quantum circuit. However, this performance came at the expense of relatively long optimization times. Our data indicates that the optimization time needed to find suitable angles for the variational quantum circuit increases exponentially with *p*, but further investigation is needed to verify this scaling.

We have seen that routing with additional capacity constraints is a difficult problem for the hybrid quantum-classical approach to handle. This becomes more evident when isolating the inequality constraint. Then we can see that the energy landscape has multiple scattered local minima. In future work, one might want to consider a different formulation for the capacity constraint^58^.

Moreover, Fig. 8 shows that the QAOA fails to distinguish solutions that satisfy all constraints but differ in cost. In approximate optimization we are not seeking the optimal solution, just any solution close to the optimum, and Fig. 8 shows that the QAOA successfully shifts the probability distribution towards lower-energy solutions. However, if there is no difference in probability between the worst solution satisfying all constraints and the best solution satisfying all constraints, the QAOA is not useful for the HVRP. The failure we observe may be due to the small energy gap between the different feasible solutions; as such, it could be circumvented by restating the problem such that feasible solutions are separated by larger energy gaps. One way of achieving this goal is to conduct a rigorous hyperparameter optimization to weight the cost and the constraints accordingly. Similar ideas were explored for the knapsack problem in Ref.^51^.

## Outlook

The classical optimization procedure is a central component in all variational algorithms and key to their success. Therefore, finding new optimizers is one interesting area of research. Several works have investigated machine-learning-based optimizers^63,71,72^. A study of such optimizers for the HVRP could boost the performance of the algorithm.

Even though we assumed a noise-free system, the performance is not competitive with modern high-performance heuristics^28,52^ which can solve instances with more than 1,000 customers. Such optimizers are not limited in the number of decision variables, but rather by the running time for the optimization. However, the problems we considered are too small for a meaningful comparison. Furthermore, the assumption of a noise-free system does not hold for NISQ computers and a decrease in performance can be expected if the algorithm is executed on such a device^11,73^. An interesting topic for future work would be to investigate the HVRP in combination with QAOA under the assumption of a noisy system. Moreover, running small problems on an actual quantum computer could give a better view on the applicability of the QAOA to the HVRP and its competitiveness with classical heuristics.

In Fig. 5, we could see that the knapsack constraint creates an energy landscape with rapidly oscillating local minima. This makes it difficult for many optimizers to find a good approximate solution. It would be interesting to investigate why this particular problem is difficult and if it is possible to relax the knapsack constraint or reformulate it such that the optimization landscape becomes easier to maneuver.

Furthermore, the QAOA can be expanded to the quantum alternating operator ansatz^74^, which recently has been applied to the CVRP^75^. Investigating different mixer Hamiltonians for the HVRP could lead to a better overall performance. Ideally, the mixer Hamiltonian would provide a framework that keeps the algorithm in the subspace of allowed solutions^76^. We can envision an extension to the so-called XY mixer that respects the constant Hamming weight in subsections of the solution bitstring. It can be noted here that the XY mixer has been investigated with noise taken into consideration^73^, and the numerical results in that work indicate that the presence of noise can cause the state to leave the subspace of allowed solutions. Even so, the algorithm might still lead to higher-quality solutions since the optimization does not need to explore every possible bit string. However, it should be noted that finding mixer Hamiltonians for general constraints has been shown to be an NP-complete problem^77^. Furthermore, both the QAOA and the quantum alternating operator ansatz have been shown to suffer from barren plateaus^78^, i.e., their optimization landscapes may become exponentially flat; it would be important to understand to what extent this is the case for the HVRP.

Finally, we note that for real-world applications it might be necessary to consider multiple depots. Therefore, another avenue to explore are more Ising formulations that allow for solving different variations of VRPs.

## Supplementary Information

Supplementary Information.

## Data Availability

Data is available from D.F. upon reasonable request.
