# Supplementary material for: Applying quantum approximate optimization to the heterogeneous vehicle routing problem
Source: Sci Rep. 2024 Oct 25;14:25415. doi: 10.1038/s41598-024-76967-w (PMC11512041; doi:10.1038/s41598-024-76967-w)
Supplement: Supplementary file 1 — Supplementary Information. [file 41598_2024_76967_MOESM1_ESM.pdf]

Supplementary Information for "Applying quantum approximate optimization to the heterogeneous vehicle routing problem"

David Fitzek,[1,2,*] Toheed Ghandriz,[2,3] Leo Laine,[2,3] Mats Granath,[4,†] and Anton Frisk Kockum[1,‡]

[1] *Department of Microtechnology and Nanoscience, Chalmers University of Technology, 412 96 Gothenburg, Sweden*

[2] *Volvo Group Trucks Technology, 405 08 Gothenburg, Sweden*

[3] *Department of Mechanics and Maritime Sciences, Chalmers University of Technology, 412 96 Gothenburg, Sweden*

[4] *Department of Physics, University of Gothenburg, 412 96 Gothenburg, Sweden*

(Dated: March 4, 2024)

ADDITIONAL INFORMATION FOR THE SIMULATION OF THE QAOA

The information used to determine the fixed and variable cost for the QAOA simulations in this work is shown in Table I. The table shows the fuel consumption and cost per 100 km, the price of the chassis, and loading capacity [1] for the two truck types we consider in Fig. 3 in the main text: a rigid truck (`rt`) and a tractor semitrailer (`ts`).

Table I. Fuel consumption f and corresponding cost c_{road} per 100 km, the price c_{chass} of the chassis as well as the loading capacity [1] for each of the two different truck types considered in the simulations of this paper. Note that the loading capacity does not take into account the size or weight of items that can be carried by the truck. The information in this table is used to determine the variable and fixed costs in Eq. (14) in the main text.

	`rt`	`ts`
f [l/100 km]	28.6 [2]	34.5 [3]
c_{road} [€/100 km][a]	34.32	41.4
c_{chass}[€]	75,000	150,000
$m_{\max}$ [items]	3	4

[a] The price per liter is taken to be 1.2 € [4].

* davidfi@chalmers.se
† mats.granath@physics.gu.se
‡ anton.frisk.kockum@chalmers.se

[1] T. Ghandriz, *Transportation Mission Based Optimization of Heavy Vehicle Fleets including Propulsion Tailoring*, Licentiate thesis, Chalmers University of Technology (2018).
[2] "Average fuel consumption in Australia," (2020).
[3] U. Clausen, H. Friedrich, C. Thaller, and C. Geiger, eds., *Commercial Transport: Proceedings of the 2nd Interdisciplinary Conference on Production Logistics and Traffic 2015*, Lecture Notes in Logistics (Springer International Publishing, 2015).
[4] "Germany Diesel prices, 09-Mar-2020," (2020).
